# The direct and accurate determination of major elements Ca, K, Mg and Na in water by HR-ICPMS

**DOI:** 10.1038/s41598-018-34028-z

**Published:** 2018-12-10

**Authors:** Kenny Nadeau, Zoltán Mester, Lu Yang

**Affiliations:** National Research Council Canada, 1200 Montreal Rd, Ottawa, K1A 0R6 Ontario Canada

## Abstract

A direct, accurate and precise method is reported for major elements Ca, K, Mg and Na measurements in river and drinking water using a high resolution ICP-MS. Double isotope dilution calibration was used for the determination of Mg whereas the combined standard addition and internal standardization (Sc) was used for Ca, K and Na measurements. The method is validated by analysis fresh water SLRS-5, SLRS-6 and SRM1640a with satisfactory results characterized by high precisions of 0.055% to 0.66% RSD (or 0.29–1.8% relative combined uncertainty) for all four analytes, superior to those reported in earlier studies. As noted, use of internal standard Sc has significantly (3–33 times) improved measurement precisions for Ca, K and Na compared to standard addition calibration alone. The proposed method was applied for the determination of major elements Ca, K, Mg and Na in a candidate drinking water CRM AQUA-1. Values of 1.908 ± 0.007 µg g^−1^, 8.27 ± 0.14 µg g^−1^, 0.660 ± 0.010 µg g^−1^ and 13.76 ± 0.05 µg g^−1^ (*u*, k = 1) were obtained for Mg, Ca, K and Na in AQUA-1 drinking water, respectively.

## Introduction

Freshwater is essential for agriculture, industry and vital to human existence^1–4^. Minerals are necessary for human life and play important roles in metabolic functions, such as maintenance of pH, osmotic pressure, muscle contraction, energy production, and in almost all other aspects of life^2,5^. Although minerals are essential to life, they could become toxic at elevated concentrations. Continuous increase in population and anthropogenic activities has led to significant increase in contaminant levels in our freshwater reservoirs, which has raised many concerns over the quality of our drinking water. Consequently, monitoring major elements such as Ca, K, Mg and Na, toxic elements such as As, Cd and Pb and other trace elements in water has become one of the most frequently performed analyses for freshwater and drinking water to ensure that their quality meets the regulatory guidelines.

For the determination of trace and ultra-trace elements from ppq to ppb levels, undoubtedly inductively couple plasma mass spectrometry (ICPMS) is the most powerful among atomic spectrometry techniques due to its high sensitivity, low detection limits and multi-element detection capability^6,7^. Traditionally, major elements Ca, K, Mg and Na at ppm levels in freshwater/drinking water have been determined by more cost efficient techniques of atomic absorption spectrometry (AAS)^5,8–11^ or inductively coupled plasma-atomic emission spectrometry (ICP-AES)^8,12–14^. Major ions of Ca, K, Mg and Na at ppm levels in freshwater/drinking are exceeding the linear dynamic range of the ICPMS instruments which employ secondary electron multiplier (SEM) detector, preventing their direct measurements. Moreover, polyatomic interferences arising from sample matrix such as ^12^C^12^C^+^ and ^48^Ca^2+^ on ^24^Mg^+^, ^12^C^13^C^+^ on ^25^Mg^+^, ^38^Ar^1^H^+^ and ^23^Na^16^O^+^ on ^39^K^+^ and ^12^C^16^O^16^O^+^ on ^44^Ca^+^ encountered in ICP-MS need to be resolved or removed for their accurate determination. Consequently, only a few publications^4,14,15^ reported using ICPMS for the determination of major ions of Ca, K, Mg and Na in freshwater water directly. Of course, samples can be diluted to bring the major ions into the linear detection range for their measurements. But diluting Na to ppb level for the measurements is hampered by high Na blank in the DIW (1–10 ppb). Alternately, ICPMS equipped with a Faraday cup detector in addition to the SEM detector^16^ could be used for the direct determination of Ca, K, Mg and Na at ppm level in fresh water. Polyatomic interferences on Mg, Ca and K isotopes can be circumvented by either using high resolution (HR) ICPMS or quadrupole qICPMS with a reaction cell to resolve potential interferences.

Moreover, if two interference-free isotopes of a given element can be found, isotope dilution (ID) can be employed, which generally provides superior accuracy and precision over other calibration strategies, including external calibration and standard addition calibration, since a ratio instead of absolute intensity measurement is used^17–19^. Certified Reference Materials (CRMs) play important role in the validation of methods used for analysis. For the certification of elements in the production of CRMs, methods which can achieve the highest accuracy and precision are desired. The objective of this study was to develop an accurate and precise method to directly measure major elements Ca, K, Mg and Na by HR-ICPMS to complement the routinely used ICP-AES method for the certification of a new drinking water CRM AQUA-1. To the best of our knowledge, this is first application of employing gravimetric sample preparation for the direct, accurate and high precision measurements of Ca, K and Na in fresh water using combined standard addition and internal standardization calibration by HR-ICPMS.

## Methods

### Instrumentation

All measurements were made with a HR-ICPMS ElementXR, Thermo Fisher Scientific (Bremen, Germany), equipped with a combination of cyclonic and Scott-type spray chambers and 50 µL min^–1^ MCN50 PFA nebulizer (Elemental Scientific, Omaha, NE, USA). A plug-in quartz torch with a quartz injector and a Pt guard electrode were used. The ElementXR is equipped with an additional Faraday cup detector in addition to the SEM detector. As a result of the combination Faraday and SEM detector, the detection range is extended from ppq to ppm (counting SEM: ~2 ppq to ~2 ppb; Analog SEM: ~10 ppt to ~1000 ppb and Faraday: ~10 ppb to ~2000 ppm)^16^.

### Reagents and solutions

High purity nitric acid was obtained by sub-boiling distillation of reagent HNO_3_ in a quartz still. The de-ionized water (DIW) was obtained from a NanoPure system (Barnstead/Thermolyne Corp, Iowa, USA). Natural isotopic abundance Ca, K, Mg and Na stock solutions of SRM 3109a, SRM 3141a, SRM 3131a and SRM 3152a at 10000 µg g^−1^ levels were obtained from National Institute of Standards and Technology (NIST, Gaithersburg, MD, USA). A 3.91 µg g^−1^ Mg standard solution used for the reverse spike isotope dilution of the enriched spike solution was prepared gravimetrically in pH 1.6 HNO_3_ solution. A mix standard solution containing 1049.7 µg g^−1^, 81.83 µg g^−1^ and 328.97 µg g^−1^ for Ca, K and Na, respectively, used for standard addition calibration was prepared by dilution of their stocks with pH 1.6 HNO_3_ solution. A 250 µg g^−1^ Sc internal standard solution was prepared from a 1000 µg g^−1^ Sc (SCP Science, Montreal, Quebec, Canada).

Enriched ^25^Mg isotope (as metal, 98%^+^, Trace Sciences International, Richmond Hill, Ontario, Canada) was dissolved in HNO_3_ and diluted with DIW. A spike solution of 4.7 µg g^−1^ was prepared by dilution of the ^25^Mg stock with pH 1.6 HNO_3_ solution.

National Research Council Canada (NRCC, Ottawa, Canada) river water CRMs SLRS-5 and SLRS-6, and NIST (Gaithersburg, MD, USA) natural water SRM1640a were used for method validation.

### Sample preparation and analysis procedure

For Mg measurements by isotope dilution calibration, 1.0 g subsamples (n = 3–7) of SLRS-5 and SLRS-6 or 2.0 g subsamples of SRM1640a were weighed into pre-cleaned polyethylene bottles. Appropriate masses of the 4.7 µg g^−1 25^Mg spike solution were added to achieve a ratio of ^24^Mg/^25^Mg near 1 and then diluted to 10 g in pH 1.6 HNO_3_ solution. Three sample blanks were prepared similarly in pH 1.6 HNO_3_ solution with addition of 10% of enriched spike used in the sample. For reverse ID, 0.30 g aliquots (n = 4) of 4.7 µg g^−1 25^Mg spike solution were accurately weighed into the pre-cleaned bottles to which each 0.48 g of a 3.91 µg g^−1^ natural abundance Mg standard solution was added to achieve ^24^Mg/^25^Mg ratio near 1. The contents were then diluted to 10 g with pH 1.6 HNO_3_ solution. A 2 µg g^−1^ Mg (natural abundance) was prepared in pH 1.6 HNO_3_ for mass bias correction.

To achieve more accurate results, gravimetric sample preparation (rather than traditional volume based sample preparation) was performed for the determination of Ca, K and Na using the combined standard addition and internal standardization. In brief, 60 g subsamples (n = 3–7) of each water CRMs were weighed into pre-cleaned bottles with addition of 0.6 g of a 250 µg g^−1^ Sc to each bottle. Four 15 g subsamples of each of the above solutions were spiked with appropriate masses of a mix working standard (1049.7 µg g^−1^, 81.83 µg g^−1^ and 328.97 µg g^−1^ for Ca, K and Na, respectively) to result in approximately a 1-, 2- and 4-fold increase in the analyte content, respectively. Appropriate masses of pH 1.6 HNO_3_ solution were added to each set of unspiked sample, spiked 1x and spiked 2x, respectively, to matching the final mass of the spiked 4x in order to achieving identical matrix for the set of samples.

Optimization of the HR-ICPMS was performed as recommended by the manufacturer. Typical operating conditions of the HR-ICPMS are given in Table 1. Triple detector cross calibration was performed daily. Low resolution (LR) was used for the Na measurements, medium resolution (MR) was used for Mg measurements and high resolution (HR) was used for Ca and K to separate polyatomic interferences on Mg, Ca and K isotopes. For the determination of Mg using double ID calibration, all samples were measured in a same sequence. The 2 2 µg g^−1^ Mg (natural abundance) was measured at the beginning of the sequence and one of the reverse ID solution was measured repeatedly between samples to monitor mass bias drift. Intensities of Mg isotopes obtained in all samples were corrected by intensities of a blank of pH 1.6 HNO_3_ solution.

**Table 1:** HR-ICPMS typical operating conditions.

Plasma Ar gas flow rate	16.0 L min^−1^
Auxiliary Ar gas flow rate	1.0 L min^−1^
Nebulizer Ar gas flow rate	1.058 L min^−1^
Sampler cone orifice (nickel)	1.00 mm
Skimmer cone orifice (nickel)	0.88 mm
Lens voltage	Focus: −975 V; x deflection: −3.60 V; y deflection: 3.30 V; Shape: 129 V; Quad1: 0 V (LR), 3.76 V (MR), 3.51 V (HR); Quad2: 0 V (LR), 1.94 V (MR), 1.91 V (HR); Focus Quad1: 0 V (LR), −0.38 V (MR), −3.63 V (HR)
SEM	1650 V
Faraday Deflection	−209 V
Dead Time	26 ns
Resolution	300, 4200 MR and 10300 HR (mass offset and lock mass features used)
Data acquisition	E-scan, 5% (LR) and 125% (MR and HR) mass window; 0.0050 s sample time, 100 (LR) and 15 (MR and HR) samples per peak; 3 runs and 50 passes; 100% integration window for LR and 80% for MR and HR

For the determination of Ca, K and Na, measurement sequence follows: unspiked sample - spiked 4x - spiked 2x - spiked 1x - unspiked sample. The blank corrected intensities of ^23^Na^+^, ^44^Ca^+^, ^39^K^+^ and ratios of ^23^Na^+^/^45^Sc^+^, ^44^Ca /^45^Sc^+^ and ^39^K^+^/^45^Sc^+^ were used for the quantitation of Ca, K and Na using either standard addition calibration alone or combined standard addition and internal standardization, respectively. Despite of the direct measurements of the river and drinking water, no significant memory effects were encountered after 30 seconds rinsing with pH 1.6 HNO_3_ between samples.

## Results

### The determination of Mg in waters using double isotope dilution method

Standard addition and isotope dilution (ID) calibrations are often used to overcome the matrix effects. In addition, ID can compensate effects due to instrument drift, and subsequent loss of analyte during sample preparation has no impact on the final results^17–19^, provided that isotopic equilibrium is achieved first. Moreover, ID provides superior measurement accuracy and precision compared to other calibration strategies. Monoisotopic elements such as Na, cannot be calibrated by ID, therefore standard addition calibration is often used. Usually poorer precision of final results is expected due to the larger uncertainty associated with linear regression of intensities used for the quantitation. Alternatively, combining the standard addition and internal standardization may significantly improve analytical precision through the correction of drifts during analysis^20–23^. In order to provide a second set of high quality data for Ca, K, Mg and Na for the certification of AQUA-1 drinking water CRM, complementary to the ICP-AES results, Mg was measured using double ID. Both Ca and K could be measured by ID, but cost of enriched spikes of Ca and K for these elements was not justifiable during this certification project. Therefore, Ca and K along with Na were measured using combined standard addition and internal standardization.

Double ID was employed for the quantitation of Mg in SLRS-5, SLRS-6 and SRM1640a waters, as well as for the candidate CRM AQUA-1 drinking water^19,23^:

1 $${w}_{x}={w}_{z}\cdot \frac{{m}_{y}}{{m}_{x}}\cdot \frac{{m}_{z}}{{m^{\prime} }_{y}}\cdot \frac{{A}_{y}-{B}_{y}\cdot {R}_{n}}{{B}_{xz}\cdot {R}_{n}-{A}_{xz}}\cdot \frac{{B}_{xz}\cdot {R^{\prime} }_{n}-{A}_{xz}}{{A}_{y}-{B}_{y}\cdot {R^{\prime} }_{n}}$$

where subscripts of x, y, and z denote sample, spike and standard, respectively; *w* is the mass fraction of Mg (µg g^−1^); *m* is the mass of sample, spike or standard solution (g); ${m^{\prime} }_{y}$ is the mass of spike used to prepare the reverse ID solution (g); *A* and *B* are abundance of ^24^Mg and ^25^Mg isotopes; *R*_*n*_ and ${R^{\prime} }_{n}$ are the measured ^24^Mg/^25^Mg ratio (mass bias corrected) in the spiked sample and reverse ID solution, respectively.

Validation of the double ID method for the determination of Mg in freshwaters was achieved by analysis three water CRMs, and results are shown in Table 2.

**Table 2:** Summary of Results for SLRS-6, SLRS-5 and SRM1640a, µg g^−1^.

Material/Analyte	Method of analysis	Measured value, µg g^−1^, *u*, k = 1 (*u*_R_)	SD (µg g^−1^), RSD, n = 3	Certified value, µg g^−1^ (U, k = 2)
SLRS-6/Ca	Std. Add. with IS	8.82 ± 0.15 (1.7%)	0.03, 0.14%	8.77 ± 0.20
	Std. Add. alone	8.72 ± 0.74 (8.5%)	0.35, 4.0%	
SLRS-5/Ca	Std. Add. with IS	10.64 ± 0.18 (1.7%)	0.05, 0.46%	10.5 ± 0.4
	Std. Add. alone	11.23 ± 0.42 (3.7%)	0.32, 2.9%	
SRM1640a/Ca	Std. Add. with IS	5.62 ± 0.10 (1.8%)	0.04, 0.66%	5.57 ± 0.016*
	Std. Add. alone	5.62 ± 0.20 (3.6%)	0.10, 1.8%	
SLRS-6/K	Std. Add. with IS	0.640 ± 0.010 (1.6%)	0.004, 0.56%	0.652 ± 0.054
	Std. Add. alone	0.663 ± 0.056 (8.4%)	0.011, 1.6%	
SLRS-5/K	Std. Add. with IS	0.842 ± 0.013 (1.5%)	0.005, 0.60%	0.839 ± 0.036
	Std. Add. alone	0.904 ± 0.036 (4.0%)	0.016, 3.0%	
SRM1640a/K	Std. Add. with IS	0.576 ± 0.009 (1.6%)	0.003, 0.60%	0.5753 ± 0.002*
	Std. Add. alone	0.583 ± 0.029 (5.0%)	0.010, 1.7%	
SLRS-6/Na	Std. Add. with IS	2.727 ± 0.008 (0.29%)	0.002, 0.055%	2.77 ± 0.22
	Std. Add. alone	2.43 ± 0.33 (13.6%)	0.044, 1.8%	
SLRS-5/Na	Std. Add. with IS	5.262 ± 0.017 (0.32%)	0.003, 0.057%	5.38 ± 0.10
	Std. Add. alone	5.23 ± 0.15 (2.9%)	0.089, 1.7%	
SRM1640a/Na	Std. Add. with IS	3.113 ± 0.012 (0.38%)	0.004, 0.12%	3.112 ± 0.031*
	Std. Add. alone	3.145 ± 0.053 (1.7%)	0.045, 1.4%	
SLRS-6/Mg	ID	2.125 ± 0.008 (0.38%)	0.003, 0.14%	2.137 ± 0.058
SLRS-5/Mg	ID	2.496 ± 0.009 (0.36%)	0.008, 0.30%	2.54 ± 0.16
SRM1640a/Mg	ID	1.029 ± 0.004 (0.39%)	0.003, 0.28%	1.0502 ± 0.003*

^*^Reference value; *u****:*** combined standard uncertainty; *u*_R_*:* relative combined standard uncertainty, %; SD: standard deviation and RSD: relative standard deviation.

### Quantitation of Ca, K and Na

During the preliminary studies, slight matrix effect was observed for these analytes in natural waters, thus standard addition calibration was chosen to obtain more accurate results. Standard addition calibration (Std. Add.) alone and the combined standard addition and internal standardization (Std. Add. with IS) were compared for the determination of Ca, K and Na in three CRMs. Sc was added to the samples as an internal standard, to compensate for matrix effects and instrument drift. Na was measured in LR using the Faraday cup detector. Both Ca and K were measured in HR to remove any possible polyatomic interferences on ^44^Ca and ^39^K isotopes with use of triple detector mode. The following equation is employed to obtain the mass fraction of analyte using combined standard addition and internal standardization based on gravimetric sample preparation^23^:

2 $$\frac{{m}_{{\rm{std}}-{\rm{i}}}\cdot {w}_{{\rm{std}}}}{{m}_{s-{\rm{i}}}}\cdot \frac{{m}_{xf}}{{m}_{x}}=b\cdot {R}_{i}+a\,and\,{w}_{x}=-\,a$$

where subscripts of *x* and std denote sample and standard, respectively; *w* is the mass fraction of the analyte (µg g^−1^); *R*_i_ is the measured intensity ratio of analyte to Sc in a set of standard addition solutions: i = 0 (unspiked sample), 1 (spiked at 1x), 2 (spiked at 2x), 4 (spiked at 4x); *m*_std-i_ is the mass of standard used to prepare the set of spiked samples (g); *m*_s−i_ is the mass of sample aliquot used to prepare the set of spiked samples; *m*_x_ and *m*_xf_ are the masses (g) of the sample before and after the addition of internal standard (g); *b* and *a* are slop and intercept, respectively.

Similarly the following equation is used to obtain the mass fraction of analyte using standard addition calibration alone^23^:

3 $$\frac{{m}_{{\rm{std}}-{\rm{i}}}\cdot {w}_{{\rm{std}}}}{{m}_{{\rm{s}}-{\rm{i}}}}\cdot \frac{{m}_{xf}}{{m}_{x}}=b\cdot {I}_{i}\cdot \frac{{m}_{s{\rm{f}}-{\rm{i}}}}{{m}_{{\rm{s}}-{\rm{i}}}}+a\,and\,{w}_{x}=-\,a$$

where subscripts of *x* and std denote sample and standard, respectively; *w* is the mass fraction of the analyte (µg g^−1^); *m*_std−i_ is the mass of standard used to prepare a set of spiked samples (g), i = 0 (unspiked sample), 1 (spiked at 1x), 2 (spiked at 2x), 4 (spiked at 4x); *m*_s-i_ is the mass of sample aliquot used to prepare the set of spiked samples; *m*_x_ and *m*_xf_ are the masses (g) of the sample before and after the addition of internal standard (g); and *I*_i_ is the measured intensity in the set of samples (i = 0, 1, 2, 4); *b* and *a* are slop and intercept, respectively.

Validation of the standard addition method for the determination of Ca, Na and K in freshwaters was achieved by analysis three water CRMs, and results are also summarized in Table 2.

### Final Quantitation of Mg, Ca, K and Na in the new CRM AQUA-1 Drinking Water

For the final determination of Mg using double ID calibration and Ca, Na and K using combined standard addition and internal standardization in AQUA-1 drinking water, 7 replicates of AQUA-1 were performed, and results are shown in Table 3.

**Table 3:** Results for Ca, K, Na and Mg in AQUA-1.

Analyte	Method of analysis	Measured value, µg g^−1^, *u*, k = 1, (*u*_R_)	SD µg g^−1^, RSD	#Replicates
Ca	Std. Add. with IS	8.27 ± 0.14 (1.7%)	0.04, 0.49%	7
K	Std. Add. with IS	0.660 ± 0.010 (1.5%)	0.005, 0.59%	7
Na	Std. Add. with IS	13.76 ± 0.05 (0.36%)	0.01, 0.07%	7
Mg	ID	1.908 ± 0.007 (0.37%)	0.004, 0.20%	7

*u****:*** combined standard uncertainty; *u*_R_***:*** relative combined standard uncertainty, %; SD: standard deviation and RSD: relative standard deviation.

Uncertainties for measured mass fractions of Ca, K, Na and Mg based on Eqs 1–3 were estimated in accordance with the JCGM 100:2008 “Guide to the Expression of Uncertainty in Measurement”^24^. The combined standard uncertainty of a measurement result *y*, designated by *u*_c_(y) can be obtained from the following Equation (4):

4 $${u}_{c}^{2}(y)=\sum _{i=1}^{N}{(\frac{\partial f}{\partial {x}_{i}})}^{2}{u}^{2}({x}_{i})+2\cdot \sum _{i=1}^{N-1}\sum _{j=i+1}^{N}(\frac{\partial f}{\partial {x}_{i}})\cdot (\frac{\partial f}{\partial {x}_{j}})\cdot u({x}_{i},{x}_{j})$$

where $y=f({x}_{1},{x}_{2},\ldots ,{x}_{N})$. ${\rm{\partial }}f/{\rm{\partial }}{x}_{i}$ are partial derivatives, *u*(x_i_) is the standard uncertainty associated with the input *x*_i_, and *u*(*x*_i_, *x*_j_) is the estimated covariance associated with *x*_i_ and *x*_j_. Uncertainties results are presented in Tables 2 and 3. Clearly combined uncertainties (*u*) are larger than the standard deviations obtained from the replicate measurements, this is because that *u* includes the contributions from all parameters during the measurement process.

## Discussion

As shown in Table 2, clearly, the measured mass fractions of Mg in SLRS-5 and SLRS-6 based on double ID method agree well with their certified values, confirming the accuracy of the proposed method. The determined value for Mg is slightly lower than the value in NIST 1640a, however the Mg value in NIST 1640a is only a reference value. A method detection limit of 0.003 µg g^−1^ based on ID calibration was obtained from three spiked blanks. High precisions, in the range 0.14 to 0.30% RSD, were achieved, superior to previously reported precisions of Mg in freshwater^5,10–12,14–16,25,26^. The above results confirm that the proposed method is suitable for the determination of Mg in the candidate drinking water CRM AQUA-1.

As shown in Table 2, overall, results obtained using the combined standard addition and internal standardization agree with certified/reference values in SLRS-5, SLRS-6 and NIST 1640a, confirming the accuracy of method used. Results obtained using the standard addition calibration alone are in general agreement with certified/reference values in SLRS-5, SLRS-6 and NIST 1640a with an exception of the Ca which is slightly higher than the value in NIST 1640a. Note that Ca value in NIST 1640a is only reference value. Reference value ranges for Ca, Na and K in NIST 1640a are quite small, possibly due to large data sets and combination of various methods used to generate the final reference values. Evidently, the use of Sc internal standard significantly improves measurement precision and accuracy. High precisions of 0.055% to 0.66% RSDs for all three analytes in three CRMs using the combined standard addition calibration with Sc internal standardization were obtained, superior to those reported in previous studies^5,10–12,14–16,24,25^ and comparable to values reported in NIST 1640a. About 3–33 fold improvements in precisions (3-fold for K and 33- for Na) were obtained using the combined standard addition calibration with Sc internal standardization, compared to measurement precisions obtained by using standard addition calibration alone. Notably, precisions of Na measurements in all three CRMs are below 0.12% RSDs using the combined standard addition calibration with Sc internal standardization, superior to precisions obtained for Mg using double ID calibration. This is possibly because Mg was measured at MR on a transit peak, whereas Na was measured at LR on a flat top peak, in which better precision of measurements is obtained. As shown in Table 2, precisions for Mg using double ID are still about 2- to 3-fold better than precisions obtained for Ca and K wherein all measurements are on transit peaks (MR or HR), demonstrating the superiority of ID method. Method detection limits of 0.0005, 0.0008 and 0.001 µg g^−1^ based on combined standard addition and Sc internal standardization are estimated from 3 SD of analyte in three blanks for Ca, K and Na, respectively, suitable for the quantitation of these analytes in water.

## Conclusions

A direct, accurate and precise determination of major elements Ca, K, Mg and Na in river and drinking water is presented based on detection by HR-ICPMS equipped with a Faraday cup and SEM detectors using double ID calibration for Mg and the combined standard addition and internal standardization for Ca, K and Na, respectively. The reported method provides accurate and precise measurements for all four analytes (0.055–0.66% RSD or 0.29–1.8% *u*_R_), superior to those reported in earlier studies^5,10–12,14–16,25,26^. As demonstrated, use of internal standard Sc has significantly improved measurement precisions for K, Ca, and Na, about 3–33 fold improvements compared to measurement precisions obtained using standard additions calibration alone. The proposed method is applicable for the direct determination of major elements in freshwaters.

## Data Availability

The datasets generated during and/or analysed during the current study are available from the corresponding author on reasonable request.
